# Clinical characteristics of allergic bronchopulmonary mycosis caused by *Schizophyllum commune*

**DOI:** 10.1002/clt2.12327

**Published:** 2023-12-31

**Authors:** Tsuyoshi Oguma, Takashi Ishiguro, Katsuhiko Kamei, Jun Tanaka, Junko Suzuki, Akira Hebisawa, Yasushi Obase, Hiroshi Mukae, Takae Tanosaki, Shiho Furusho, Koji Kurokawa, Kentaro Watai, Hiroto Matsuse, Norihiro Harada, Ai Nakamura, Takuo Shibayama, Rie Baba, Kentaro Fukunaga, Hisako Matsumoto, Hisano Ohba, Susumu Sakamoto, Shinko Suzuki, Shintetsu Tanaka, Takahiro Yamada, Akira Yamasaki, Yuma Fukutomi, Yoshiki Shiraishi, Takahito Toyotome, Koichi Fukunaga, Terufumi Shimoda, Satoshi Konno, Masami Taniguchi, Katsuyoshi Tomomatsu, Naoki Okada, Koichiro Asano, Japan ABPM Research Program

**Affiliations:** ^1^ Division of Pulmonary Medicine Department of Medicine Tokai University School of Medicine Kanagawa Japan; ^2^ Department of Respiratory Medicine Saitama Cardiovascular and Respiratory Center Saitama Japan; ^3^ Division of Clinical Research Medical Mycology Research Center Chiba University Chiba Japan; ^4^ Department of Infectious Diseases Ishinomaki Red Cross Hospital Miyagi Japan; ^5^ Department of Respiratory Medicine National Hospital Organization Tokyo National Hospital Tokyo Japan; ^6^ Department of Respiratory Medicine Nagasaki University Graduate School of Biomedical Sciences Nagasaki Japan; ^7^ Division of Pulmonary Medicine Department of Medicine Keio University School of Medicine Tokyo Japan; ^8^ Department of Respiratory Medicine Kanazawa Municipal Hospital Kanazawa Japan; ^9^ Clinical Research Center National Hospital Organization Sagamihara National Hospital Kanagawa Japan; ^10^ Division of Respirology Department of Internal Medicine Toho University Ohashi Medical Center Tokyo Japan; ^11^ Department of Respiratory Medicine Juntendo University Faculty of Medicine and Graduate School of Medicine Tokyo Japan; ^12^ Department of Respiratory Medicine National Hospital Organization Okayama Medical Center Okayama Japan; ^13^ Pulmonary Division Internal Medicine Saiseikai Utsunomiya Hospital Tochigi Japan; ^14^ Division of Respiratory Medicine Department of Internal Medicine Shiga University of Medical Science Shiga Japan; ^15^ Department of Respiratory Medicine Graduate School of Medicine Kyoto University Kyoto Japan; ^16^ Department of Respiratory Medicine National Hospital Organization Tenryu Hospital Shizuoka Japan; ^17^ Department of Respiratory Medicine Toho University Omori Medical Center Tokyo Japan; ^18^ Department of Respiratory Medicine Suwa Central Hospital Nagano Japan; ^19^ Department of Respiratory Medicine Yokosuka Municipal Hospital Kanagawa Kanagawa Japan; ^20^ Department of Respiratory Medicine Matsushita Memorial Hospital Osaka Japan; ^21^ Division of Respiratory Medicine and Rheumatology Faculty of Medicine Department of Multidisciplinary Internal Medicine Tottori University Tottori Japan; ^22^ Department of Veterinary Medicine Obihiro University of Agriculture and Veterinary Medicine Obihiro Japan; ^23^ Clinical Research Center Fukuoka National Hospital Fukuoka Japan; ^24^ Faculty of Medicine Department of Respiratory Medicine Hokkaido University Sapporo Japan

**Keywords:** allergic bronchopulmonary aspergillosis, allergic bronchopulmonary mycosis, *Aspergillus*, asthma, *Schizophyllum commune*

## Abstract

**Background:**

Allergic bronchopulmonary mycosis (ABPM) is an allergic disease caused by type I and type III hypersensitivity to environmental fungi. *Schizophyllum commune*, a basidiomycete fungus, is one of the most common fungi that causes non‐*Aspergillus* ABPM.

**Objective:**

Herein, we attempted to clarify the clinical characteristics of ABPM caused by *S. commune* (ABPM‐Sc) compared with those of allergic bronchopulmonary aspergillosis (ABPA).

**Methods:**

Patients with ABPM‐Sc or ABPA were recruited from a nationwide survey in Japan, a multicenter cohort, and a fungal database at the Medical Mycology Research Center of Chiba University. The definition of culture‐positive ABPM‐Sc/ABPA is as follows: (1) fulfills five or more of the 10 diagnostic criteria for ABPM proposed by Asano et al., and (2) positive culture of *S. commune/Aspergillus* spp. in sputum, bronchial lavage fluid, or mucus plugs in the bronchi.

**Results:**

Thirty patients with ABPM‐Sc and 46 with ABPA were recruited. Patients with ABPM‐Sc exhibited less severe asthma and presented with better pulmonary function than those with ABPA (*p* = 0.008–0.03). Central bronchiectasis was more common in ABPM‐Sc than that in ABPA, whereas peripheral lung lesions, including infiltrates/ground‐glass opacities or fibrotic/cystic changes, were less frequent in ABPM‐Sc. *Aspergillus fumigatus*‐specific immunoglobulin (Ig)E was negative in 10 patients (34%) with ABPM‐Sc, who demonstrated a lower prevalence of asthma and levels of total serum IgE than those with ABPM‐Sc positive for *A. fumigatus*‐specific IgE or ABPA.

**Conclusions:**

Clinical characteristics of ABPM‐Sc, especially those negative for *A. fumigatus*‐specific IgE, differed from those of ABPA.

## 1 INTRODUCTION

Allergic bronchopulmonary aspergillosis (ABPA) is an allergic airway disease clinically characterized by asthmatic symptoms, hypersensitivity to *Aspergillus fumigatus* or other *Aspergillus* species, and radiographic findings, such as mucus plugs in the bronchi and central bronchiectasis. Environmental fungi other than *Aspergillus* spp. can cause similar pathologies, designated allergic bronchopulmonary mycosis (ABPM). Diagnosis of non‐*Aspergillus* ABPM is often difficult because of the lack of appropriate serological tests and atypical clinical phenotypes. Among the 17 cases with biopsy‐proven ABPM reported by Ishiguro et al.,^1^ 7 (41%) lacked any predisposing conditions such as asthma or cystic fibrosis, 7 (41%) lacked peripheral blood eosinophilia, and 3 (18%) presented with total serum immunoglobulin (Ig) E levels lower than 1000 IU/mL. All of which are required for the diagnosis of ABPA/ABPM with diagnostic criteria proposed by the International Society for Human and Animal Mycology (ISHAM).^2^, ^3^

Chowdhary et al. reviewed case reports on non‐*Aspergillus* ABPM and reported that *Schizophyllum commune* was the third most frequent fungus causing non‐*Aspergillus* ABPM, following *Candida* and *Bipolaris* spp.^4^ *S. commune* is a basidiomycetous fungus with a small size of its conidia (3–4 × 1–1.5 μm) and a relatively high temperature for germination (30–35°C), which makes *S. commune* optimal for colonization in the lower airways that is required for the development of ABPM.^5^, ^6^ Since we reported the first case of ABPM caused by *S. commune* (ABPM‐Sc) in 1994,^7^ most cases of ABPM‐Sc have been reported in Japan. *S. commune* was the most common non‐*Aspergillus* filamentous fungus isolated from sputum and bronchial specimens in a nationwide survey of ABPA/ABPM in Japan.^8^

The clinical features of ABPM‐Sc have not yet been well characterized due to the difficulty of its diagnosis.^8^ Recently, we established new diagnostic criteria that show better sensitivity (90.5%) for non‐*Aspergillus* ABPM than that of the Rosenberg–Patterson (14.3%) or ISHAM criteria (45.6%).^9^ Furthermore, we identified glucoamylase as a major allergen of *S. commune* (Sch c 1) and developed an enzyme‐linked immunosorbent assay (ELISA) to measure *S. commune*‐specific IgG and IgE to evaluate type I and III allergies to *S. commune*.^10^ With these inventions in mind, we attempted to identify the clinical characteristics of ABPM‐Sc in the present study.

## 2 METHODS

### 2.1 Participants of ABPM‐Sc and ABPA

Physician‐diagnosed cases of ABPM‐Sc with positive culture of *S. commune* in sputum, bronchial lavage fluid, or mucus plugs in the bronchi were surveyed among the registered cases in the nationwide study of ABPA/ABPM in Japan^8^ or in the fungal disease database at the Medical Mycology Research Center of Chiba University. Clinical data were retrospectively collected from the physicians in a case review meeting held in November 2018 and/or the questionnaires described below. ABPA cases culture‐positive for *Aspergillus* spp. were selected from our multicenter prospective registry of ABPA, ABPM, or related diseases performed at 14 clinical centers in Japan between 2013 and 2019.

The questionnaire asked about age, sex, age at ABPA/ABPM‐Sc onset, comorbidities, such as physician‐diagnosed asthma, and its severity. The treatment steps required to control asthma were reported according to the updated version of the 2016 statement by the Global Initiative for Asthma (GINA).^11^ Laboratory data including peripheral blood eosinophil counts; total serum IgE levels; serological tests including IgE, IgG, and precipitins specific for *A. fumigatus* or *S. commune*; skin tests for fungal antigens; fungal cultures in sputum and bronchial samples; pulmonary functions including forced vital capacity (FVC), forced expiratory volume in 1 s (FEV_1_), FEV_1_/FVC, and thoracic computed tomography (CT) findings including central bronchiectasis, mucus plugs, and high attenuation mucus (HAM) in the central airways; infiltrates/ground‐glass opacity (GGO); and fibrotic/cystic changes in the peripheral lungs were obtained from medical charts.

These studies were approved by the institutional review board of Tokai University Hospital (#13R‐107, #18R‐291). The patients selected from our multicenter prospective cohort study of ABPA/ABPM provided written informed consent. For the other patients, informed consent was obtained from the Tokai University Hospital website as an opt‐out. Patients who declined to participate in the study were excluded.

### 2.2 Diagnosis of culture‐positive ABPA or ABPM‐Sc

Among the cases of physician‐diagnosed ABPM‐Sc/ABPA selected as described, we analyzed the cases that fulfilled five or more of the 10 components in the diagnostic criteria for ABPM proposed by Asano et al.^9^ and were culture‐positive for either *S. commune* or *Aspergillus spp*. The diagnostic criteria^9^ included the following items: (1) current or previous history of asthma or asthmatic symptoms, (2) peripheral blood eosinophil ≥500 cells/μL, (3) total serum IgE ≥417I U/mL, (4) immediate cutaneous hypersensitivity or specific IgE for filamentous fungi, (5) presence of precipitins or specific IgG for filamentous fungi, (6) filamentous fungal growth in sputum cultures or bronchial lavage fluid, (7) presence of fungal hyphae in bronchial mucus plugs, (8) central bronchiectasis on CT, (9) presence of mucus plugs in the central bronchi, based on CT/bronchoscopy or history of mucus plug expectoration, and (10) HAM in the bronchi on CT. Patients who met six or more of these diagnostic criteria were diagnosed with definite ABPM, and those who met five items were diagnosed with probable ABPM.

The modified Rosenberg–Patterson (Table S1) and ISHAM criteria (Table S2) were used to evaluate the sensitivity and specificity for diagnosing culture‐positive ABPM‐Sc and ABPA.^2^, ^3^, ^9^ Regarding the Rosenberg–Patterson criteria, a case fulfilling all seven criteria was defined as definitive ABPM, and a case fulfilling six criteria except for central bronchiectasis was considered probable ABPM. For the ISHAM criteria, a patient who met the criteria in the absence of predisposing conditions such as asthma or cystic fibrosis was considered probable ABPM.

### 2.3 Measurement of fungus‐specific IgE, IgG, and precipitins

*S. commune*‐specific IgE and IgG titers were measured using ELISA as previously reported.^10^ Briefly, sera from the patients were incubated at 25°C for 2 h in a well coated with a recombinant *S. commune*‐derived glucoamylase (Sch c 1) synthesized using *Escherichia coli* expression system. After the wells were washed with Tris‐buffered saline with 0.1% Tween‐20 (TBS‐T, Bio‐Rad Laboratories), goat anti‐human IgG antibody conjugated with horseradish peroxidase (HRP) (MP Biomedicals) or mouse anti‐human IgE antibody conjugated with biotin and streptavidin‐HRP (MP Biomedicals) 10,000 or 3500‐fold diluted in TBS‐T was applied to detect *S. commune*‐specific IgE or IgG antibodies, respectively. Following incubation at 37°C for 1 h and washing with TBS‐T, HRP substrates were added and the absorbance of the wells was measured at 450 nm. Values greater than two standard deviations above the mean of healthy patients were considered positive. An inhibition assay was performed to determine whether Sch c 1 cross‐reacted with *A. fumigatus*‐specific IgE in the sera from ABPM patients sensitized to *A. fumigatus* (Supporting Information S1).

*A. fumigatus*‐specific IgE concentrations were measured using the ImmunoCAP system (Thermo Fisher Scientific). *A. fumigatus*‐specific IgE ≥0.35 U_A_/mL was defined as positive. *A. fumigatus*‐specific IgG was determined using the ImmunoCAP system or complement fixation method. The levels of *A. fumigatus*‐specific IgG (ImmunoCAP) > 60 mg_A_/L for patients aged <55 years and 45 mg_A_/L for those aged ≥55 years were considered positive.^12^ Precipitating antibodies in the serum were evaluated by Ouchterlony double immunodiffusion testing using crude extracts of *A. fumigatus* and *S. commune.*

### 2.4 Statistical analysis

Numerical data were presented as the median and interquartile range (IQR), and categorical data were presented as numbers and percentages. Categorical variables were compared using Fisher's exact test, while continuous variables were compared using the Mann–Whitney *U*‐test or Kruskal–Wallis test, followed by the Bonferroni/Dunn procedure as a post hoc analysis. Statistical analyses were performed using International Business Machine (IBM) Statistical Package for the Social Sciences (SPSS) Statistics ver. 24 (IBM) and GraphPad Prism 5 (GraphPad Software). The results were two‐sided, and a *p*‐value < 0.05 was considered statistically significant.

## 3 RESULTS

A total of 38 patients were recruited as physician‐diagnosed ABPM‐Sc, among whom 33 met the diagnostic criteria for culture‐positive ABPM‐Sc in the present study. There were 46 patients in the multicenter cohort who met the criteria for culture‐positive ABPA. Three cases of ABPM‐Sc were also culture‐positive for *A. fumigatus* (Table S3) and were excluded from further analysis. Therefore, 30 cases of ABPM‐Sc and 46 cases of ABPA were analyzed. Seven cases of ABPM‐Sc have been reported elsewhere as case reports.^13^, ^14^, ^15^, ^16^, ^17^, ^18^, ^19^

The clinical characteristics of the patients with ABPM‐Sc and ABPA are shown in Table 1. The median age at onset of ABPM‐Sc was 59 years, which was not significantly different from that of ABPA (67 years). Both ABPM‐Sc and ABPA developed more frequently in women than in men (67% and 59%, respectively). The prevalence of asthma in the ABPM‐Sc group was lower than that in the ABPA group, but the difference was not statistically significant (50% vs. 70%, *p* = 0.10). Two cases of ABPM‐Sc had COPD, but there were no cases with other predisposing conditions such as cystic fibrosis or tuberculosis. There was a significant difference in the severity of asthma (*p* = 0.008); more than two‐thirds of ABPM‐Sc‐associated asthma (71%) were mild (GINA step 1 or 2), whereas most patients with asthma coexisting with ABPA were moderate to severe (73%). There were no differences in the peripheral blood eosinophil counts or total serum IgE levels between the ABPM‐Sc and ABPA groups. *S. commune*‐specific IgE levels were measured in 12 patients with ABPM‐Sc, among whom 11 patients (92%) presented with positive *S. commune*‐specific IgE. In the ABPA group, 45 patients (98%) were positive for *A. fumigatus*‐specific IgE and one patient was negative for specific IgE but positive for skin test. Interestingly, *A. fumigatus*‐specific IgE was also positive in 19 cases (66%) of ABPM‐Sc among 29 cases, excluding one untested case. IgG or precipitating antibodies for *S. commune* were positive in 11 (85%) of 13 patients with ABPM‐Sc, whereas those for *A. fumigatus* were positive in 40 (87%) cases of ABPA. FEV_1_ and FEV_1_/FVC were higher in patients with ABPM‐Sc (*p* = 0.008–0.03). Central bronchiectasis was observed significantly more frequently in ABPM‐Sc (83%) than that in ABPA (61%, *p* = 0.04), whereas infiltrate/GGO and fibrotic/cystic changes in the lungs were less common in ABPM‐Sc (*p* = 0.14 and *p* = 0.02, respectively). Sensitivity analysis of ABPM‐Sc cases positive for *S. commune*‐specific IgE (*n* = 11, Table S4) demonstrated similar differences in clinical characteristics such as the severity of asthma, pulmonary function, and CT findings from the case of ABPA, although some differences did not reach the statistical significance.

**TABLE 1:** Demographic and laboratory data of the patients with ABPM‐Sc and ABPA.

	ABPM‐Sc	ABPA	*p* ^a^
*n* (%)	30	46	−
Age at onset of ABPM, *y*	59 (43–69)	67 (51–71)	0.10
Women, *n* (%)	20 (67)	27 (59)	0.63
Asthma, *n* (%)	15 (50)	32 (70)	0.10
Age at onset, years	27 (9–50)	32 (12–50)	0.94
Treatment step (1–2/3–5, *n* [%])	10/4 (71/29)	8/22 (27/73)	0.008
Duration between onset of asthma and ABPA, years	11 (3–42)	30 (11–41)	0.12
Laboratory data at diagnosis			
Peripheral blood eosinophil counts (/μL)	655 (381–1162)	983 (472–1505)	0.15
Serum IgE levels (IU/mL)	2020 (471–5797)	1954 (530–4583)	0.92
*A. fumigatus*‐specific IgE, U_A_/mL	0.80 (0.10–3.34)	11.20 (3.29–30.05)	<0.001
*A. fumigatus*‐specific IgG, mg_A_/L	NA	68.5 (28.8–125.8)	
Positive serological tests			
*A. fumigatus*‐specific IgE, *n* (%)	19 (66)^b^	45 (98)	<0.001
*A. fumigatus*‐specific precipitin/IgG, *n* (%)	7 (30)^c^	40 (87)	<0.001
*S. commune*‐specific IgE, *n* (%)	11 (92)^d^	NA	
*S. commune*‐specific precipitin/IgG, *n* (%)	11 (85)^e^	NA	
Pulmonary function test			
FVC, %predicted	94 (86–106)	99 (82–113)	0.99
FEV_1_, %predicted	98 (77–109)	80 (65–92)	0.008
FEV_1_/FVC, %	77 (72–81)	70 (61–80)	0.03
Thoracic computed tomography findings			
Central bronchiectasis, *n* (%)	25 (83)	28 (61)	0.04
Mucus plugs, *n* (%)	28 (93)	40 (87)	0.47
High attenuation mucus, *n* (%)	22 (76)	27 (59)	0.14
Infiltration/GGO, *n* (%)	24 (80)	43 (94)	0.14
Fibrotic/cystic change, *n* (%)	0 (0)	8 (17)	0.02

*Note*: Values are medians (interquartile range) or the proportion of patients in each study group, if not otherwise specified.

Abbreviations: ABPA, allergic bronchopulmonary aspergillosis; ABPM‐Sc, ABPM culture positive for *S. commune*; FEV_1_, forced expiratory volume in 1 s; FVC, forced vital capacity; GGO, ground‐glass opacity; Ig, immunoglobulin; NA, not assayed.

^a^ Between the two groups.

^b^ *n* = 29.

^c^ *n* = 23.

^d^ *n* = 12.

^e^ *n* = 13.

**FIGURE 1:**
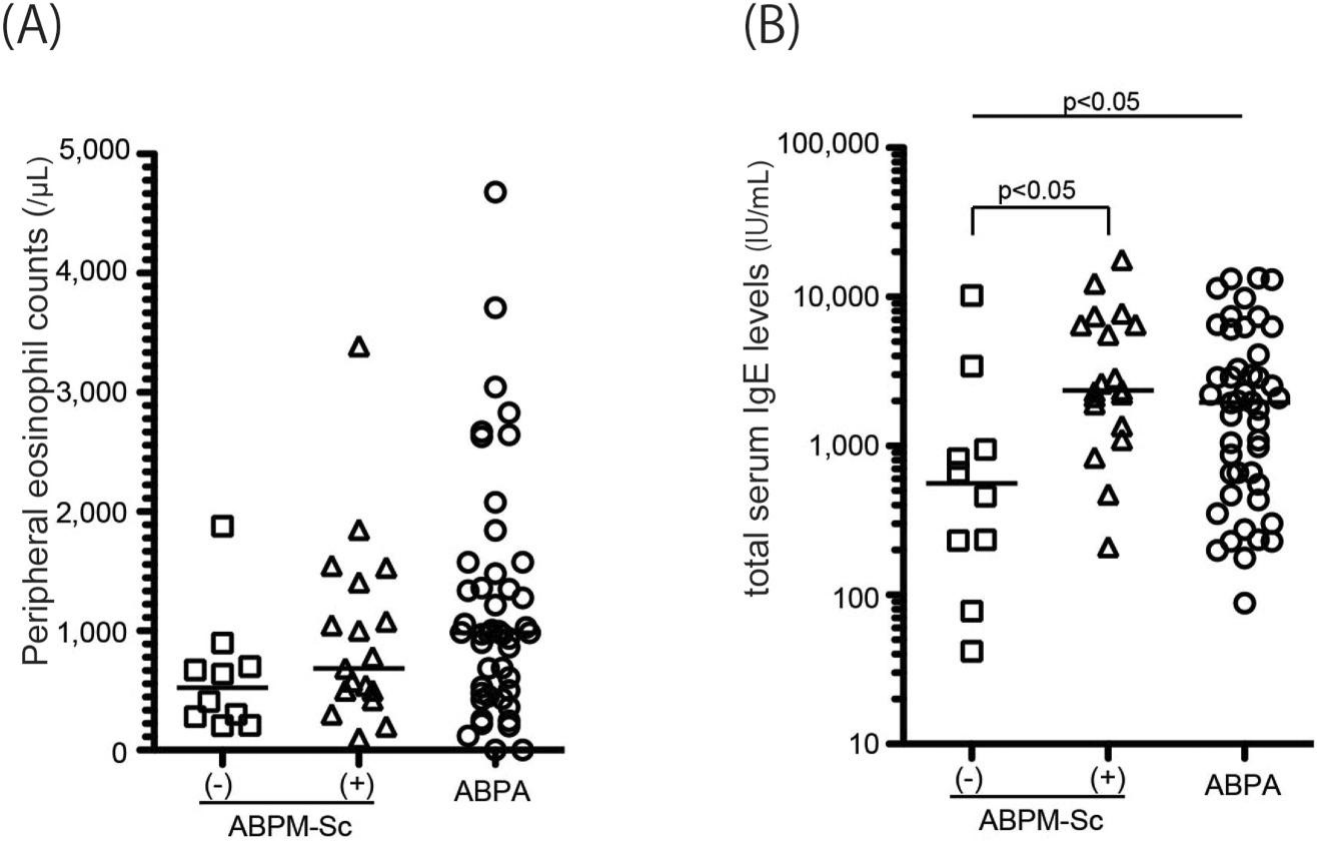
Peripheral eosinophil counts (A) and total serum IgE levels (B) among the cases of ABPM‐Sc negative or positive for *A. fumigatus*‐specific IgE (*n* = 10 and 19, respectively) and ABPA (*n* = 46). The number of cases with less than 1000 IU/mL in total IgE concentration was 8 (80%), 3 (16%), and 17 (37%) in ABPM‐Sc negative or positive for *A. fumigatus*‐specific IgE and ABPA, respectively. ABPM‐Sc, ABPM culture positive for *S. commune*; ABPA, allergic bronchopulmonary aspergillosis; Ig, immunoglobulin.

We found that there were two subgroups in the patients with culture‐positive ABPM‐Sc: sensitized and not sensitized to *A. fumigatus*. Therefore, we compared the clinical characteristics of the three groups: the ABPM‐Sc cases negative or positive for *A. fumigatus*‐specific IgE, and the cases of ABPA (Table 2). Patients with ABPM‐Sc negative for *A. fumigatus*‐specific IgE (*n* = 10) were less likely to be accompanied by asthma (30%) than those with ABPM‐Sc positive for *A. fumigatus*‐specific IgE (*n* = 19, 58%) or ABPA (70%). Although there were no differences in peripheral blood eosinophil counts between *A. fumigatus*‐specific IgE‐negative and positive cases of ABPM‐Sc (Figure 1A), median total IgE level was significantly lower in the *A. fumigatus*‐specific IgE‐negative cases of ABPM‐Sc (562 IU/mL) than that in the positive cases (2366 IU/mL, *p* < 0.05) or in the ABPA cases (1954 IU/mL, *p* < 0.05, Figure 1B). There was no difference between *A. fumigatus*‐specific IgE‐positive and ‐negative cases of ABPM‐Sc in the age of onset, sex, and thoracic CT images. However, FEV_1_ was significantly different among the three groups (*p* = 0.02), with the lowest values observed in patients with ABPA (80% of predicted value), followed by the cases of ABPM‐Sc positive (93%) and negative (103%) for *A. fumigatus*‐specific IgE. We performed a similar analysis comparing the clinical characteristics of ABPM‐Sc with asthma (*n* = 15) and without asthma (*n* = 15, Table S5). However, we did not observe any differences between them.

**TABLE 2:** Comparison of demographic and laboratory data between cases of ABPA and ABPM‐Sc either positive or negative for *Aspergillus*‐specific IgE.

*A. fumigatus*‐specific IgE	ABPM‐Sc		ABPA	p*
	Negative *n* = 10	Positive *n* = 19	*n* = 46	
Age at onset of ABPM, *y*	65 (44–77)	57 (38–66)	67 (51–71)	0.11
Women	7 (70)	12 (63)	27 (59)	0.84
Asthma*	3 (30)	11 (58)	32 (70)	0.07
Treatment step (1–2/3–5, *n* [%])	3/0 (100/0)	6/4 (60/40)	8/22 (27/73)	0.01
Laboratory data at diagnosis				
Peripheral blood eosinophil counts (/μL)	523 (263–750)	686 (500–1409)	983 (472–1505)	0.12
Serum IgE levels* (IU/mL)	562*** (194–1571)	2366 (1363–6504)	1954 (530–4583)	0.03
*A. fumigatus*‐specific IgE*, U_A_/mL	0.09**^,^*** (0.05–0.16)	3.13 (0.85–5.66)	11.20 (3.29–30.05)	<0.001
*A. fumigatus*‐specific precipitin/IgG‐positive, *n* (%)	1**^a^ (17)	6**^b^ (38)	40 (87)	<0.001
*S. commune*‐specific IgE‐positive, *n* (%)	5^a^ (83)	6^a^ (100)	NA	
*S. commune*‐specific precipitin/IgG‐positive, *n* (%)	6^c^ (86)	5^a^ (83)	NA	
Thoracic computed tomography				
Central bronchiectasis, *n* (%)	9 (90)	16 (84)	28 (61)	0.08
Mucus plugs, *n* (%)	10 (100)	17 (90)	40 (87)	0.76
High attenuation mucus, *n* (%)	7 (78)	14 (74)	27 (59)	0.43
Infiltration/GGO, *n* (%)	7 (70)	16 (84)	43 (94)	0.08
Fibrotic/cystic change, *n* (%)	0 (0)	0 (0)	8 (17)	0.08
FEV_1_, %predicted*	103 (83–112)	93 (74–107)	80 (65–92)	0.02

*Note*: Values are medians (interquartile range) or the proportion of patients in each study group, if not otherwise specified.

Abbreviations: ABPA, allergic bronchopulmonary aspergillosis; ABPM‐Sc, ABPM caused by *S. commune*; FEV_1_, forced expiratory volume in 1 s; GGO, ground‐glass opacity; Ig, immunoglobulin.

^a^ *n* = 6.

^b^ *n* = 16.

^c^ *n* = 7.

*Among the three groups, **p < 0.05, versus ABPA, ***p < 0.05 versus ABPM‐Sc with sensitization to *A. fumigatus*.

We examined the sensitivity of the Rosenberg–Patterson and ISHAM criteria for the diagnosis of ABPA and ABPM‐Sc (Table 3). The sensitivity for the diagnosis of ABPA was 39% with the Rosenberg–Patterson criteria and 87% with the ISHAM criteria, which was compatible with our previous analysis. In contrast, the sensitivity for the diagnosis of ABPM‐Sc was significantly lower than that of ABPA, 13% (*p* = 0.02) with the Rosenberg–Patterson criteria and 27% (*p* < 0.001) with the ISHAM criteria. In cases with measurement of *S. commune*‐specific IgE and IgG, the diagnostic sensitivity of ABPM‐Sc with ISHAM criteria increased to 67%, suggesting the clinical importance of these tests (Table 3). Even in the absence of *S. commune*‐specific IgE and IgG measurements, a definite diagnosis could be made in 72% of ABPM‐Sc cases according to Asano's criteria.

**TABLE 3:** Sensitivity with the diagnostic criteria for ABPM.

	*n*	Asano	Rosenberg–Patterson		ISHAM	
		Definite	Definite	Definite/probable	Definite	Definite/probable
ABPM‐Sc, *n* (%)	30	25 (83)	3 (10)	4 (13)	5 (17)	8 (27)
*A. fumigatus*‐specific IgE						
Positive, *n* (%)	19	14 (74)	3 (16)	3 (16)	3 (16)	5 (26)
Negative, *n* (%)	10	10 (100)	0 (0)	1 (10)	2 (20)	3 (30)
*S. commune*‐specific IgE/IgG						
Measured, *n* (%)	12	12 (100)	3 (25)	4 (33)	5 (42)	8 (67)
Not measured, *n* (%)	18	13 (72)	0 (0)	0 (0)	0 (0)	0 (0)
ABPA, *n* (%)	46	43 (94)	12 (26)	18 (39)	27 (59)	40 (87)

Abbreviations: ABPA, allergic bronchopulmonary aspergillosis; ABPM‐Sc, ABPM caused by *S. commune*; Ig, immunoglobulin; ISHAM, International Society for Human and Animal Mycology.

Figure S1 demonstrates the treatment of patients with ABPM‐Sc (Figure S1A) and ABPA (Figure S1B). In the patients with ABPM‐Sc, seven (23%) were treated with oral corticosteroids alone, eight (27%) were treated with anti‐fungal agents alone, and nine (30%) were treated with both. Itraconazole was the commonly used anti‐fungal agent for ABPM‐Sc, except for one patient who was treated with voriconazole. In addition, an anti‐interleukin‐5 (IL‐5) receptor‐alpha antibody, benralizumab, was used in two cases in combination with oral corticosteroids and itraconazole. Six patients were observed without any medication, and three showed improvements within a month after removing the mucus plug by bronchoscopy or discarding the contaminated air conditioner.^19^ The other three patients also improved gradually only with inhaled corticosteroids.

## 4 DISCUSSION

To date, this is the largest study of ABPM‐Sc in which we could clarify its unique clinical characteristics and phenotypes. Compared with culture‐positive ABPA, ABPM‐Sc is accompanied by milder asthma and better pulmonary function. Central bronchiectasis is more common in ABPM‐Sc, whereas lung parenchymal lesions such as infiltrates/GGO or fibrotic/cystic changes are less frequent. There seem to be two phenotypes of ABPM‐Sc: one co‐sensitized to *A. fumigatus*. and the other not co‐sensitized. The not co‐sensitized phenotype was less allergic, with a lower prevalence of asthma and lower total serum IgE levels.

*S. commune* inhabits anywhere in the world except in extremely cold places^5^, ^6^; cases of fungal sinusitis have been reported worldwide, such as in France or India.^20^, ^21^ In contrast, cases of ABPM‐Sc have been reported almost exclusively in Japan since the first report in 1994.^7^ There are several reasons why ABPM‐Sc cases have rarely been identified. First, there were no serological tests for *S. commune*‐specific IgE or IgG levels. For the diagnosis of non‐*Aspergillus* ABPM, diagnostic criteria for ABPA have been modified and applied; however, these criteria require a positive skin test or precipitin that requires *S. commune* extracts not commercially available in the absence of *S. commune*‐specific IgE and IgG tests. Second, in contrast to the diagnosis of allergic fungal rhinosinusitis, which is based on the detection of fungi in the surgically resected specimen, previous diagnostic criteria for ABPA/ABPM have downgraded the role of fungal culture.^22^ Third, *S. commune* might have been overlooked in the laboratory as a miscellaneous fungus because of the non‐specific appearance of colonies on culture. Fourth, as observed in the present study, many cases of ABPM‐Sc lack either asthma or elevated total serum IgE levels. Chowdhary et al. speculated that the predominance of ABPM‐Sc cases in Japan was due to the awareness of this disease rather than geographical or climatic factors.^23^ The usefulness of the new diagnostic criteria and the development and standardization of ELISA for *S. commune*‐specific IgE and IgG would enhance the awareness of ABPM‐Sc in other regions of the world.^9^, ^10^ In addition, we recently identified volatile organic compounds specifically produced by *S. commune*, which may improve the detection rate of *S. commune* in culture.^24^

Two‐thirds of the cases with ABPM‐Sc presented with *A. fumigatus*‐specific IgE in the present study. Cross‐reactivity to crude allergen extracts from different fungi is often observed^25^, ^26^ although it is still unknown whether *S. commune* contains antigens that cross‐react with *A. fumigatus*. Toyotome reported that specific IgG for Sch c 1 was positive in 2 of 10 patients with aspergilloma or chronic pulmonary aspergillosis, suggesting the possibility of cross‐reactivity between *Aspergillus* spp. and *S. commune*.^10^ However, Sch c 1 did not inhibit *A. fumigatus*‐specific IgE ELISA (Figure S2), suggesting that there is limited cross‐reactivity between *A. fumigatus* and Sch c 1. Another possibility is that some patients with ABPM‐Sc who were sensitized to *S. commune* had also been exposed and sensitized to *A. fumigatus* simultaneously or non‐simultaneously. Ishiguro demonstrated a case in which there was no intersection of the respective lines of *S. commune* and *A. fumigutus* in Ouchterlony double immunodiffusion testing, suggesting that the patient was sensitized to both fungi.^17^ Several case reports have demonstrated that *A. fumigatus* and *S. commune* are simultaneously colocalized in the airways.^25^, ^26^ There were three cases of ABPM in this study, in which *A. fumigatus* and *S. commune* were identified in the same bronchial specimen (Table S3). Therefore, it is important to consider the possibility that the causative fungi of ABPM change during the course, as previously reported.^17^, ^19^, ^27^ Identification of fungal in the mucus plugs obtained by fiberoptic bronchoscopy, in combination with serological tests, would be helpful to determine the fungi responsible for ABPM.

The patients with ABPM‐Sc who were also sensitized to *A. fumigatus* demonstrated highly allergic phenotypes as observed in those with ABPA, such as more frequent and severe asthma, higher total serum IgE, or more frequent demonstration of infiltration/GGO in CT. It has been demonstrated that *A. fumigatus* is highly allergenic and induces higher IgE production than other fungi.^28^ In contrast, there was no difference in the clinical characteristics of ABPM‐Sc between patients with and without asthma (Table S5). Therefore, co‐sensitization to *A. fumigatus* may be associated with the enhanced‐allergic phenotype of ABPM‐Sc.

Standard treatment for ABPM‐Sc has not yet been established. Systemic corticosteroids and anti‐fungal agents are often used for the treatment of ABPM‐Sc.^14^, ^16^ Ishiguro et al. reported six patients with ABPM‐Sc in whom anti‐fungal agents were used as monotherapy, and a favorable response was observed in four of them (66.7%).^14^ Itraconazole, which shows a low minimum inhibitory concentration (MIC) for *S. commune* in vitro,^5^, ^29^ was used most frequently in a previous and present survey for the treatment of ABPM‐Sc with high efficacy.^14^ Voriconazole also showed low MIC for *S. commune* in vitro^5^, ^29^ and was reported to be useful for itraconazole‐refractory ABPM‐Sc cases.^14^ Six patients with ABPM‐Sc (20%) did not receive any pharmacotherapy in the present study, probably due to the lack of severe respiratory symptoms; however, irreversible destruction of bronchoparenchymal structures, such as bronchiectasis, is not uncommon for ABPM‐Sc. To avoid these consequences, early therapeutic intervention should be considered even in cases with few symptoms.

In conclusion, the clinical characteristics of ABPM‐Sc, particularly those *A. fumigatus*‐specific IgE‐negative, were different from those of ABPA. Awareness of this condition would be further enhanced with the introduction of *S. commune‐*IgE and IgG assays and new diagnostic criteria for ABPM.

## AUTHOR CONTRIBUTIONS

Tsuyoshi Oguma had full access to all of the data in the study and takes responsibility for the integrity of the data and the accuracy of the data analysis, contributed to the conception and design of the study, acquisition of data, analysis and interpretation of data, and drafting the manuscript. Takashi Ishiguro, Katsuhiko Kamei, Jun Tanaka, Junko Suzuki, Akira Hebisawa, Yasushi Obase, Hiroshi Mukae, Takae Tanosaki, Shiho Furusho, Koji Kurokawa, Kentaro Watai, Hiroto Matsuse, Norihiro Harada, Ai Nakamura, Takuo Shibayama, Rie Baba, Kentaro Fukunaga, Hisako Matsumoto, Hisano Ohba, Susumu Sakamoto, Shinko Suzuki, Shintetsu Tanaka, Takahiro Yamada, Akira Yamasaki, Takahito Toyotome, Koichi Fukunaga, Terufumi Shimoda, Satoshi Konno, Masami Taniguchi, Katsuyoshi Tomomatsu, and Naoki Okada contributed to the data entry, data interpretation, refining the study methodology, and manuscript review. Yoshiki Shiraishi and Yuma Fukutomi contributed to the conduct and analysis of the immunoassay and review of the manuscript. Koichiro Asano contributed to the conception and design of the study, the supervision of all activities related to the conduct of the study, study idea, statistical analysis, discussion, writing, and final approval of the manuscript, and served as the guarantor of the paper.

## CONFLICT OF INTEREST STATEMENT

The authors have no conflicts of interest to declare.

## Supporting information

Supporting Information S1

Figure S1

Figure S2

## Data Availability

The data that support the findings of this study are available from the corresponding author upon reasonable request.
