# Supplementary material for: Clinical characteristics of allergic bronchopulmonary mycosis caused by Schizophyllum commune
Source: Clin Transl Allergy. 2023 Dec 31;14(1):e12327. doi: 10.1002/clt2.12327 (PMC10758016; doi:10.1002/clt2.12327)
Supplement: Supplementary file 1 — Supporting Information S1 [file CLT2-14-e12327-s002.docx]

**Supplemental materials**

**Clinical characteristics of allergic bronchopulmonary mycosis caused by *Schizophyllum commune***

Tsuyoshi Oguma^a^, Takashi Ishiguro^b^, Katsuhiko Kamei^c,d^, Jun Tanaka^a^, Junko Suzuki^e^, Akira Hebisawa^e^, Yasushi Obase^f^, Hiroshi Mukae^f^, Takae Tanosaki^g^, Shiho Furusho^h^, Koji Kurokawa^h^, Kentaro Watai^i^, Hiroto Matsuse^j^, Norihiro Harada^k^, Ai Nakamura^k^, Takuo Shibayama^l^, Rie Baba^m^, Kentaro Fukunaga^n^, Hisako Matsumoto^o^, Hisano Ohba^p^, Susumu Sakamoto^q^, Shinko Suzuki^r^, Shintetsu Tanaka^s^, Takahiro Yamada^t^, Akira Yamasaki^u^, Yuma Fukutomi^i^, Yoshiki Shiraishi^a^, Takahito Toyotome^c,v^, Koichi Fukunaga^g^, Terufumi Shimoda^w^, Satoshi Konno^x^, Masami Taniguchi^i^, Katsuyoshi Tomomatsu^a^, Naoki Okada^a^, Koichiro Asano^a^, and Japan ABPM Research Program

**Supplemental methods**

*A. fumigatus*-specific IgE Inhibition Analysis

An inhibition assay was performed to determine whether *A. fumigatus*-specific IgE in the sera from ABPM patients sensitized to *A. fumigatus* cross-reacts with other allergens*.*

*A. fumigatus* extract (2 μg dry weight/well, ITEA Inc., Tokyo, Japan) was applied to a 96-well half area ELISA plate (#675061, Greiner Bio-One Co., Tokyo Japan) and incubated overnight at 4°C. Diluted pooled sera from five *A. fumigatus*-sensitized patients with ABPM were incubated with serial dilution (0.064 - 200 µg/mL) of Sch c 1, Asp f 1 (RayBiotech, GA, USA), Der p 1 (RayBiotech), or crude extracts of *A. fumigatus*, *Alternaria alternata*, and *Dermatophagoides pteronissinus* (ITEA Inc.) overnight at 4ºC. The sera were applied and incubated for 4 h at room temperature on the plate pre-blocked with 1% bovine serum albumin for 2 h at 37°C. After several wash, diluted (1:25) biotin-conjugated anti-human IgE monoclonal antibody (BioLegend, CA, USA) was added, followed by incubation for 4 h at room temperature. The plate was washed, incubated with TMB Chromogen Solution (Thermo Fisher Scientific K.K., Japan), and then the reactions were stopped with 0.5M sulfuric acid. Optimal density was measured at 450 nm and 540 nm using a microplate reader (Molecular Devices, CA, USA).

**Table S1. Modified Rosenberg**–**Patterson’s diagnostic criteria**

1. Episodic bronchial obstruction (asthma)
2. Peripheral blood eosinophilia (≥ 500/mm^3^)
3. Immediate skin reactivity or specific IgE antibody to filamentous fungal antigen
4. Precipitating antibodies or IgG antibodies against filamentous fungal antigen
5. Elevated serum IgE concentrations (≥ 417 IU/mL)
6. History of pulmonary infiltrates (transient or fixed)
7. Central bronchiectasis

**Table S2. Modified ISHAM diagnostic criteria**

Predisposing conditions

asthma, cystic fibrosis

Obligatory criteria (both should be present)

1. Immediate cutaneous hypersensitivity or elevated IgE levels against filamentous fungi
2. Elevated serum IgE concentrations (≥ 1,000 IU/mL)

Other criteria (at least two of three)

1. Precipitating antibodies or IgG antibodies against filamentous fungal antigen
2. Radiographic pulmonary opacities consistent with ABPA
3. Peripheral blood eosinophilia (≥ 500/mm^3^)

If the patient meets all other criteria, an IgE value < 1,000 IU/mL may be acceptable.

**Table S3. Characteristics of the patients positive in culture for both *A. fumigatus and S. commune***

| No. | Onset age of ABPM  /Sex | Asthma | Eo^†^ | IgE^‡^ | IgE/ IgG^§^ | | Colonizing fungi at diagnosis |
| --- | --- | --- | --- | --- | --- | --- | --- |
|  |  |  |  |  | *A. fumigatus* | *S. commune* |  |
|  | 62/F | + | 844 | 600 | -/- | NA/NA | *S.commune*  *A. fumigatus* |
|  | 61/F | + | 3,814 | 82 | -/- | NA/+ | *S.commune*  *A. fumigatus* |
|  | 53/M | + | 300 | 6,183 | +/+ | NA/+ | *S. commune*  *A. fumigatus* |

^†^Peripheral blood eosinophil counts (/L), ^‡^serum IgE levels (IU/mL), ^§^specific IgE/precipitin or IgG-positive.

NA, not analyzed.

**Table S4. Demographic and laboratory data of the patients with definite ABPM-Sc and ABPA**

|  | Definite ABPM-Sc | Definite ABPA | *P*^*^ |
| --- | --- | --- | --- |
| n (%) | 11 | 46 | - |
| Age at onset of ABPM, y | 64（52–70） | 67 (51–71) | 0.72 |
| Women, n (%) | 8 (73) | 27 (59) | 0.50 |
| Asthma, n (%) | 6 (55) | 32 (70) | 0.48 |
| Age at onset, years | 42 (20–53) | 32 (12–50) | 0.43 |
| Treatment step (1–2/3–5, n [%]) | 4/1 (80/20) | 8/22 (27/73) | 0.04 |
| Duration between onset of asthma and ABPA, years | 5 (2–30) | 30 (11–41) | 0.09 |
| Laboratory data at diagnosis |  |  |  |
| Peripheral blood eosinophil counts (/μL) | 686 (500–1,002) | 983 (472–1,505) | 0.34 |
| Serum IgE levels (IU/mL) | 948 (664–5,580) | 1,954 (530–4,583) | 0.79 |
| *A. fumigatus*-specific IgE, U_A_/mL | 0.25 (0.08–2.78) | 11.20 (3.29–30.05) | <0.001 |
| *A. fumigatus*-specific IgG, mg_A_/L | NA | 68.5 (28.9–125.8) |  |
| Positive serological tests |  |  |  |
| *A. fumigatus*-specific IgE, n (%) | 6 (55) | 45 (98) | 0.001 |
| *A. fumigatus*-specific precipitin/IgG, n (%) | 3 (43) ^†^ | 40 (87) | 0.02 |
| *S. commune*-specific IgE, n (%) | 11 (100) | NA |  |
| *S. commune*-specific precipitin/IgG, n (%) | 9 (82) | NA |  |
| Pulmonary function test |  |  |  |
| FVC, %predicted | 99 (92–127) | 99 (82–113) | 0.43 |
| FEV_1_, %predicted | 105 (93–127) | 80 (65–92) | 0.001 |
| FEV_1_/FVC, % | 80 (77–83) | 70 (61–80) | 0.02 |
| Thoracic computed tomography findings |  |  |  |
| Central bronchiectasis, n (%) | 9 (82) | 28 (61) | 0.30 |
| Mucus plugs, n (%) | 11 (100) | 40 (87) | 0.59 |
| High attenuation mucus, n (%) | 10 (91) | 27 (59) | 0.08 |
| Infiltration/GGO, n (%) | 9 (82) | 43 (94) | 0.24 |
| Fibrotic/cystic change, n (%) | 0 (0) | 8 (17) | 0.33 |

Values are medians (interquartile range) or the proportion of patients in each study group, if not otherwise specified. ^†^n = 7. *between the two groups

ABPM-Sc, ABPM culture positive for *S. commune*; ABPA, allergic bronchopulmonary aspergillosis; FEV_1_, forced expiratory volume in 1 s; FVC, forced vital capacity; GGO, ground-glass opacity; Ig, immunoglobulin; NA, not assayed.

**Table S5. Comparison of demographic and laboratory data between ABPM-Sc cases with and without asthma**

|  | ABPM-Sc | |  | |
| --- | --- | --- | --- | --- |
| Asthma | (-)  n = 15 | (+)  n = 15 | *P* |  |
| Age at onset of ABPM, y | 63 (45–75) | 53 (38–66) | 0.16 | |
| Women | 9 (60) | 11 (73) | 0.70 | |
| Laboratory data at diagnosis |  |  |  | |
| Peripheral blood eosinophil counts (/μL) | 500 (300–899) | 781 (500–1,547) | 0.09 | |
| Serum IgE levels (IU/mL) | 2,130  (234–6,448) | 1,911  (821–5,580) | 0.60 | |
| *A. fumigatus*-specific IgE, U_A_/mL | 0.25  (0.05–4.64) | 0.90  (0.36–3.50) | 0.25 | |
| Positive serological tests |  |  |  | |
| *A. fumigatus*-specific IgE, n (%) | 8 (53) | 11^†^ (79) | 0.25 | |
| *A. fumigatus*-specific precipitin/IgG, n (%) | 3^‡^ (23) | 4^§^ (40) | 0.65 | |
| *S. commune*-specific IgE, n (%) | 5^¶^ (83) | 6^¶^ (100) | 1.00 | |
| *S. commune*-specific precipitin/IgG, n (%) | 6^$^ (86) | 5^¶^ (83) | 1.00 | |
| Thoracic computed tomography |  |  |  | |
| Central bronchiectasis, n (%) | 13 (87) | 12 (80) | 1.00 | |
| Mucus plugs, n (%) | 15 (100) | 13 (87) | 0.49 | |
| High attenuation mucus, n (%) | 11 (78) | 11 (73) | 1.00 | |
| Infiltration/GGO, n (%) | 11 (73) | 13 (87) | 0.65 | |
| Fibrotic/cystic change, n (%) | 0 (0) | 0 (0) | - | |
| FEV_1_, %predicted^*^ | 104 (83–113) | 89 (75–103) | 0.20 | |

Values are medians (interquartile range) or the proportion of patients in each study group, if not otherwise specified. ^†^n = 14, ^‡^n = 13, ^§^n=10, ^¶^n=6, ^$^n=7.

ABPM-Sc, ABPM caused by *S. commune*; ABPA, allergic bronchopulmonary aspergillosis; FEV_1_, focused expiratory volume in 1 s; GGO, ground-glass opacity; Ig, immunoglobulin.

**Figure S1.** Treatment of ABPM-Sc (A) and ABPA (B)

The Venn diagram shows the numbers of patients and percentage in parenthesis who were untreated or treated with oral corticosteroids, anti-fungal agents, or biologics.

**Figure S2.**

IgE inhibition analysis using A. fumigatus extract as solid phase in pooled serum from five *A. fumigatus*-sensitized patients with ABPM.

AFE, *A. fumigatus* extract; AAE, *Alternaria alternata* extract; DPE, *Dermatophagoides pteronissinus*.
