# Supplementary figures and images for: Clinical characteristics of allergic bronchopulmonary mycosis caused by Schizophyllum commune
Source: Clin Transl Allergy. 2023 Dec 31;14(1):e12327. doi: 10.1002/clt2.12327 (PMC10758016; doi:10.1002/clt2.12327)

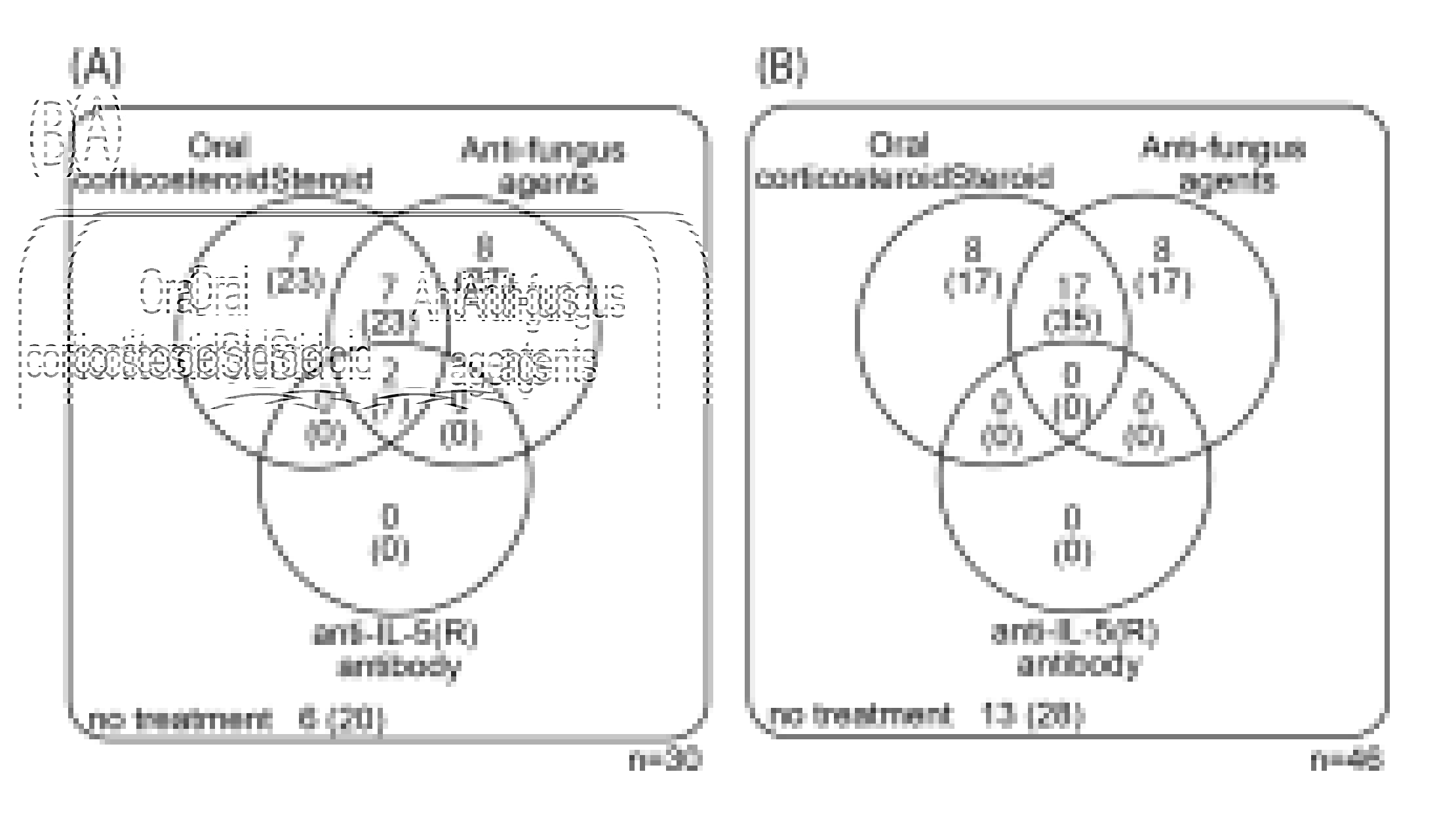

Supplement: Supplementary file 2 — Figure S1 [file CLT2-14-e12327-s001.jpeg]

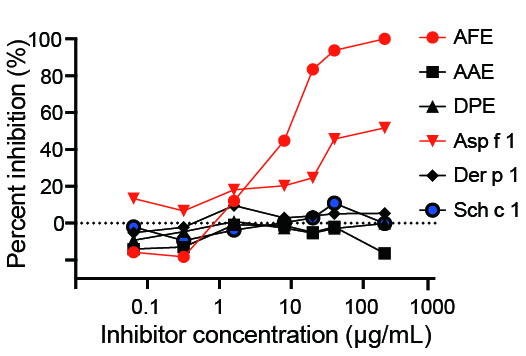

Supplement: Supplementary file 3 — Figure S2 [file CLT2-14-e12327-s003.jpg]
